# Evaluation of Feed Strategies and Changes of Stocking Rate to Decrease the Carbon Footprint in a Traditional Cow-Calf System: A Simulation Model

**DOI:** 10.3389/fvets.2021.587168

**Published:** 2021-06-09

**Authors:** Paula Toro-Mujica

**Affiliations:** Instituto de Ciencias Agroalimentarias, Animales y Ambientales (ICA3), Universidad de O'Higgins, San Fernando, Chile

**Keywords:** simulation model, carbon footprint, feed strategies, stocking rate, additive

## Abstract

One of the main production challenges associated with climate change is the reduction of carbon emissions. Increasing the efficiency of resource utilization is one way to achieve this purpose. The modification of production systems through improved reproductive, genetic, feed, and grazing management practices has been proposed to increase technical–economic efficiency, even though the “environmental viability” of these modifications has not always been evaluated. The objective of this study was to evaluate the use of feeding and management strategies on the carbon footprint (CF) and economic variables in the traditional cow–calf system in southern Chile using a simulation model. The modifications evaluated corresponded to combinations of stocking rate, use of creep feeding practices with different supplementation levels, and the incorporation of feed additives to the supplement, using factorial experiments. Additionally, the scenarios were evaluated with and without carbon sequestration. The CF for the baseline scenarios was 12.5 ± 0.3 kg of CO_2−eq_/kg of live weight (LW) when carbon sequestration was considered and 13.0 ± 0.4 kg of CO_2−eq_/kg of LW in the opposite case. Changes in stocking rate, supplementation level, and consideration of carbon sequestration in pasture and soil had a significant effect on the CF in all simulated scenarios. The inclusion of additives in the supplement did not have a significant effect on production costs. With regard to reducing greenhouse gas (GHG) emissions, incorporating canola oil presented the best average results. The model developed made the selection of environmentally viable feed strategies or management adaptations possible.

## Introduction

Livestock is one of the main agricultural activities responsible for the anthropogenic emissions of greenhouse gas (GHG). These GHG emissions from global livestock supply chains reach ~7 gigatonnes annually. Of this, 65% are associated with cattle production (1). Modifying livestock systems is a strategy to increase technical and economic efficiency. At the same time, interest exists in decreasing GHG. However, obtaining precise measurements or estimations is the first barrier to such a challenge. The Intergovernmental Panel on Climate Change (IPCC) has generated empirical methodologies with different degrees of precision (Tiers) for estimating GHG for livestock production systems through mathematical equations (2). On the other hand, more recent researchers, such as studies carried out by Cottle and Eckard (3), Niu et al. (4), and Patra and Lalhriatpuii (5), proposed models for beef cattle, dairy cattle, and small ruminants, respectively. Cottle and Eckard (3), through a meta-analysis applied to the MitiGate database of methane-mitigation studies, determined an equation that predicts the methane yield in beef cattle. The equation included the following variables: measurement method, breed, diet, and country. Niu et al. (4) used the fitting linear mixed models to estimate methane emissions, including variables, such as dry matter (DM) intake, dietary composition, milk production and composition, and body weight. Patra and Lalhriatpuii (5) developed linear and non-linear statistical models for small ruminants to predict enteric methane emissions from goats, using the variables dietary nutrient composition, intake of nutrients and energy, digestibility of energy, and organic matter.

Undoubtedly, diet and various associated nutritional management strategies are fundamental variables in any enteric methane emission estimation model. At the same time, it is one of the main routes to increase the efficiency of the livestock production systems (6–10). Then, feeding systems, pastoral resource management (11, 12), and a comprehensive perspective (13) are research areas that have been studied to reduce GHG emissions. Within nutritional management, various research studies have addressed the inclusion of additives to the diet. The proposed additives include nitrates, lipids (e.g., canola and soybean oil), monensin, yeasts, plant extracts (e.g., oregano, green tea, tannins, and saponins tea), seaweed, propolis extract, and mixtures of them. In most of these (14–20), in grazing or confined animals, a decrease in enteric methane emissions has been observed. Evaluating the effects of these changes in the general management and some nutritional management of livestock farms has been estimated through simulation models. Lurette et al. (21) created a model to evaluate the sensitivity of pastoral dairy systems to scenarios of seasonal biomass production variability. White et al. (22) used a whole-farm model to study the relative importance of reproduction, genetics, and nutritional management on minimizing the environmental impact of meat production systems. Furthermore, Rotz et al. (23) developed a simulation model to quantify environmental footprints for beef production systems in the United States. However, the use of simulation models to estimate the effect of the inclusion of feed additives in livestock production cycles, both at the level of total emissions and at the level of economic variables', is scarce (24). Then, as Legesse et al. (25) pointed out, a need exists to include evaluation of the contribution of various feeding systems to GHG emissions that can be covered through simulation models.

In southern Chile, where more than 63% of the cattle production is located, a steady reduction in the number of farms and cattle heads has occurred in recent years (26). In this area, a predominance of cow–calf systems exists, generally within small farms with low technical and economic efficiency (9). The animals are usually kept under extensive grazing regimens. This is supplemented during the winter with hay or silage. The products of these systems correspond to calves of 6–8 months old that are sold for finishing on other farms. In search of increasing the environmental viability of the system, the objective of this study was to evaluate the use of feeding and management strategies on the carbon footprint (CF) and economic variables in the traditional cow–calf system in southern Chile using a simulation model.

## Materials and Methods

### Study Area and Production System

The southern zone of Chile is constituted by three regions: La Araucanía, Los Ríos, and Los Lagos. The Region of La Araucanía has the second-largest cattle population, reaching 0.7 million heads, representing 17% of the country's cattle (24). This zone of Chile is between 38° and 39°30′S latitude and covers an area of 31,842.3 km^2^. The cattle farms are small to medium size, with an average herd of 17.7 heads of cattle (standard deviation: 81.2 cattle heads), and where 97% of farmers own < 100 animals (24). Feeding is based on natural and sown grasslands, and some crops cover nearly 10% of the total area (9, 27).

The region's climate is temperate rainy, with an average temperature of 11.2°C, an average rainfall of ~1,200 mm, and 57% of the precipitation between May and August. Forage production varies between 2 and 8 tons/ha, depending on the type of pasture, level of fertilization, and management of grazing. Forage production is seasonal, concentrating between 50 and 60% of production in the spring. This distribution forces farmers to conserve forage as hay or silage to supplement the animal diet during winter and summer (9). In addition, the seasonality of forage production determines the seasonality of the bovine production. The calving season is concentrated between the end of winter and the beginning of spring (28). This ensures the best fit between the supply and demand of forage. Thus, the breeding season is carried out in November and December (29). In the traditional cow–calf production system, the production cycle lasts between 16 and 20 months, depending upon the length of the breeding season and the weaning age. The conventional product of the current breeding systems corresponds to steer calves and heifer calves of 6–8 months with weights of 180–240 kg. Typical stocking rates in the zone studied are around 0.5–1.4 livestock units/ha/year. These stocking rates demonstrate that the systems tend to be extensive. The cow–calf system uses natural or sown grassland, and some farmers have supplementary crops. The most common supplementary crops are corn silage, oats, and alfalfa. The supplementation of cows during the winter months (June to August) is a common practice, as well as the supplementation during the breeding months.

### Limits of the Systems

**Figure 1:**
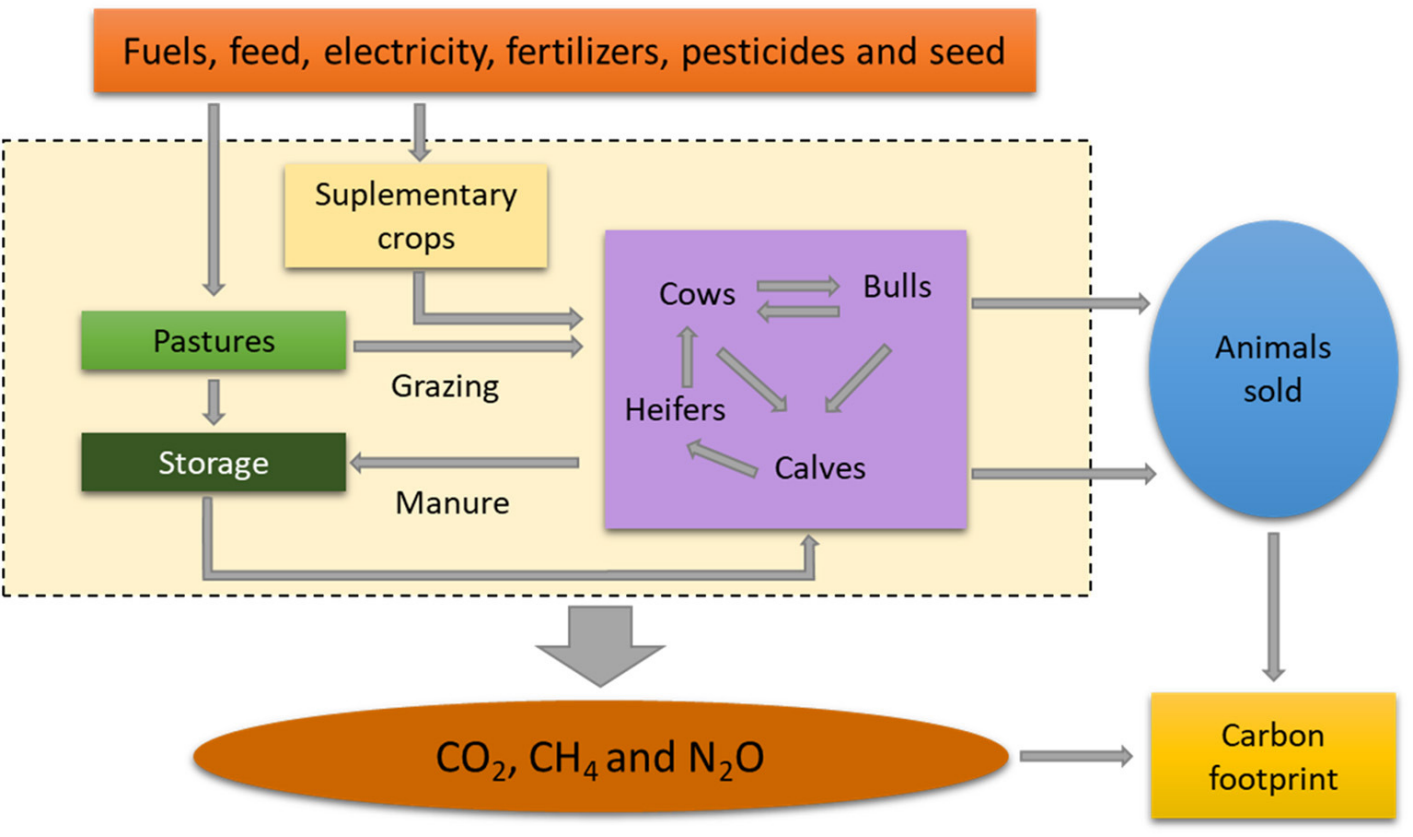
System boundaries, greenhouse gas (GHG) sources, and storage (sinks) of the cow–calf system.

The cradle-to-farm-gate methodology (30) utilized by Toro-Mujica et al. (31), Sykes et al. (32), and Cerri et al. (33) was used to estimate the system CF. This methodology involves assessing the emissions associated with all the inputs and processes within a production cycle. This evaluation is carried out until the animal leaves the farm. Therefore, emissions related to transport, slaughterhouse, livestock fairs, or other farms were not included (34–36). According to Rotz et al. (36), these activities represent <1% of the total energy used. Emissions associated with veterinary and reproductive inputs were not considered (37). Emissions related to inputs required for the production of forage and supplements, such as seed, fertilizers, pesticides, and fuel expenditures on machinery, were assigned to the DM production of the respective crop (Figure 1). Soil carbon sequestration, an ecosystem service, was estimated for soils and pastures (38–41). Quantification of the CF was calculated per kg live weight (LW) of cattle sold.

### Model Description

A simulation model was developed in Excel 2016, using spreadsheets and macros of VBA based on the model proposed by Catrileo et al. (9) to evaluate management modifications in cow–calf systems on temperate pastures in Chile. The model contains an initial data sheet where the user defines the characteristics of the farm, including size and general management. With regard to general management, fertility percentage, replacement rate, and cows and calf mortality were also included. For feed management, the model has a configuration sheet where the quantity and type of supplement are defined month by month. The model allows monthly selection and set supplementation for cows and calves. In the case of calves, supplementation is simulated through the creep feeding system. From the model, it is assumed that the farm has three pasture paddocks. In each, the type of pasture present must be selected. Subsequently, the user must select month by month which paddock will be used. The model has a database where information about nutrient composition, the CF of supplements and pasture, climatic data, and price of products, among others, is stored. The model makes daily estimates for forage intake and energy requirements, based on the equations proposed by CSIRO (42, 43) and AFRC (44). A random component was incorporated in the variables voluntary forage intake, milk production, and cow and calf mortality to obtain a stochastic model. The normal reverse distribution was used to incorporate randomness into the variables for voluntary forage intake and milk production, with a standard deviation of 7 and 7.5%, respectively (9).

A variation of Bernoulli's test was used to incorporate randomness into the variable mortality. Then, each animal was considered as an independent test (45), generating for each of them a random number with uniform distribution (0–100). When this number was less than or equal to the mortality variable, it meant that the animal has died.

#### Pastures and Supplements

The pastures used to simulate the model were tall fescue with subterranean clover (28). Data for monthly growth rates (kg DM/ha), digestibility of DM (%), and crude protein (%) were available. The inputs required for sowing and fertilization were measured in terms of CF based on Edwards-Jones et al. (46) and Saunders and Barber (47). For animals' supplementation with corn silage, energy concentration of 2.5 Mcal/kg DM, 7.5% crude protein, and 72% digestibility of DM (48) were used. The CF of the corn silage was assigned a value of 0.2 kg CO_2−eq_/kg, as reported by Adom et al. (49). Emissions attributable to additives and their transportation were not included since the low inclusion percentage does not significantly modify the CF of the supplement.

#### Carbon Sequestration

As pointed out by Toro-Mujica et al. (31), carbon sequestration was assumed to be 10% of the carbon deposited in the soil by fertilization (39) over a time period of 100 years (38). In the case of organic matter deposited in the soil by senescence of the pasture, it was assumed that 15% of the organic matter was converted into the organic matter found in soils (50).

#### Estimate of the Carbon Footprint

For CF, the original estimation algorithm of the model was complemented with the Tier 2 equations proposed by the IPCC (2, 51) to assess GHG emissions. These equations relate gross energy intake to the production of methane. Carbon dioxide (CO_2_), methane (CH_4_), and nitrous oxide (N_2_O) emissions were transformed into CO_2_ equivalents (CO_2_-eq) based on the conversion factors (1 kg of CO_2_-eq equal to 1 kg of CO_2_, 25 kg of CO_2−_eq equal to 1 kg of CH_4_, and 298 kg of CO_2_-eq equal to 1 kg of N_2_O) as proposed by the IPCC (2, 46).

The emissions converted into CO_2−eq_ were added and divided by total LW (kilogram) sold out of the farm gate (culled cows and weaned claves) to calculate CF assuming kg LW at the farm gate as the functional unit (52). The selection of this functional unit was based on the possibility of comparing the results with those of other studies.

## Baseline Simulated Scenarios

The baseline scenario corresponded to the traditional cow–calf production system (Group I) identified by Toro-Mujica et al. (53). The definition of a cow–calf production system within the simulation model includes the month of the production cycle initiation, stocking rate, age, weight of cows, pasture areas, type and time of use of the pastures, cow fertility, duration of lactation, and cow and calf mortality, as well as data referring to the use of inputs for grassland fertilization. In the actual system, the herd consisted of cows of different ages and weights; but in the model, average weight and age were used. In the baseline scenario, a stocking rate of 0.7 livestock units (LU/ha), a production cycle of 20 months, the supplementation of cows for 90 days in winter (June to August), and 90 days of postpartum were chosen. The supplementation for cows was carried out with corn silage (2 kg DM per cow per day). The calves were not supplemented.

## Proposed Feed Strategies and Experimental Scenarios

The simulation model flexibility allows using combinations of many variables. However, in order to simplify the interpretation of the results, only three variables were evaluated in the experiment. The variables included stocking rate, use of creep feeding with five levels of supplementation (0, 0.25, 0.5, 0.75, and 1 kg DM/calf/day) with corn silage from 2 months of age to age of sale, and use of feed additives in the corn silage for feeding calves (monensin with a dose of 30 mg/kg DM and canola oil with a dose of 46 g/kg DM) (17, 54).

The effect of incorporating feed additives was carried out based on the results reported by Beauchemin and McGinn (17) and Ranga Niroshan Appuhamy et al. (54). The authors suggested that the inclusion of monensin reduced methane emissions from 9 to 15% per unit of gross energy intake. On the other hand, the reduction due to the addition of canola oil was 21% per unit of gross energy intake. With regard to the cost of adding monensin to corn silage, an increase of 3% was estimated over the initial price due to its cost and its mixture with the supplement. In the case of canola oil, the price increase of the supplement was 13%.

The dimensional input variables, management variables, and the levels used in the variables' stocking rate, feed additives, and supplementation to simulate are shown in Table 1. Table 1 also includes the inputs (urea, seed, diesel, and pesticides) associated with the maintenance of the improved pasture.

**Table 1:** Input data.

**Variables**	**Value**
Beginning of breeding season (month)^a^	December
Stocking rate (LU/ha)	0.4, 0.55, 0.7, 0.85, 1
Cows (number)	59, 82, 105, 128, 150
Initial weight (kg)	414
Average age (months)	36
Weaning age (months)	6
Fertility (%)^b^	95
Replacement heifers (% of cows)	20
Native pasture production (kg DM/ha)	5,400
Native pasture area (ha)	82
Improved pasture production (kg DM/ha)	6,700
Improved pasture area (ha)	27
Initial pasture availability (kg DM/ha)	3,000
Soil organic matter (%)	8.4
Supplementation age of calves	2 months to sold
Feed additives types	Monensin and canola oil
Calves supplementation (kg DM/calf/day)	0, 0.25, 0.5, 0.75, 1
Type of supplement of calves	Corn silage
Cows supplementation (kg DM/cow/day)	2
Cows supplementation (months)	June to August/December to February
Monthly cow mortality (%)	0.5
Monthly calf mortality (birth to weaning, %)	1
Urea (kg/ha)	100
Seed use (kg/ha)	24
Diesel (kg/ha)	30
Pesticides (l/ha)	2

a *With a duration of 2 months*.

b *Pregnant cows/total mated cows*.

Data were analyzed using R software (55). All the experimental scenarios were analyzed with and without consideration of carbon sequestration in pasture and soil in order to evaluate if the variation of CF depends on the incorporation of this factor. The results were evaluated using multifactorial ANOVA (56). Analysis of multiple comparisons of means was conducted through Tukey's honestly significant difference (HSD) test or the Kruskal–Wallis test, after proof equality of variances with Levene's test. The analyses were performed with two data sets:

Baseline stocking rate (0.7 LU/ha) scenario, modified through the calf supplementation and additive uses.

Baseline stocking rate (0.7 LU/ha) scenario, modified through the change of stocking rate, the calf supplementation, and additive uses.

Since the model is stochastic, 20 simulations (reps) were run for each variable's combinations. The number of replicates required for the stochastic model was tested by the methodology described by Toro-Mujica et al. (20).

## Results

### Baseline Scenario

The CF in the baseline scenario was 12.5 ± 0.3 kg CO_2−eq_/kg of LW when carbon sequestration was not considered. The incorporation of carbon sequestration increased the CF to 13.0 ± 0.3 kg CO_2−eq_/kg of LW sold (Table 2). The sale weight and weaning of the calves was 181.5 ± 3.1 kg with 6 months of age. The weight of the cows at weaning was 542.2 ± 10.0 kg. Calves' supplementation had a significant effect on the variables CF, total emissions, weight of calves, and total kg of calves sold (*p* < 0.01, Table 2). The decrease in CF reached 4% for supplementation scenarios due to the 10.2% increase in the sale weight of calves compared with the non-supplementation scenario. With regard to the total emissions, the use of the supplement increased the total CO_2_ emissions by 2.5%. However, due to increased total production (kilos sold), the value of the CF decreased. The inclusion of additives to corn silage had a significant effect on the CF, methane emissions, and total CO_2_ emissions (Table 2). Thus, the scenarios that used monensin decreased CF by 1.6%, methane emissions by 1.7%, and total CO_2_ emissions by 1.4%, compared with the scenarios without additive. The effect produced by the addition of canola oil was slightly higher, reaching values of 2.4, 3.0, and 2.5% for CF, methane emissions, and total CO_2_ emissions, respectively.

**Table 2:** Simulated carbon footprint (CF), methane emissions, total emissions, and productive variables according to calf supplementation (creep feeding) and additives (monensin and canola oil) used in the baseline scenario (stocking rate of 0.7 LU/ha).

**Variable**	**Level**	**Carbon footprint** **(kg CO**_****2−eq****_**/kg LW)**		**Methaneemissions (tons of CO_**2−eq**_)**	**Total emissions (tons of CO_**2−eq**_)**	**Calves weight (kg LW)**	**Cows weight(kg LW)**	**Total production (kg LW)**
		**NCS**	**WCS**					
Supplementation (kg DM/calf/day)	0^1^	12.5 ± 0.3^d^	13.0 ± 0.32^d^	199.5 ± 2.1^a^	236 ± 2.4^a^	182 ± 2.6^a^	541.9 ± 10.2	10,334 ± 448^a^
	0.25	12.4 ± 0.3^c^	13.0 ± 0.32^d^	199.9 ± 2.1^a^	237.8 ± 2.4^b^	185.7 ± 2.6^b^	542 ± 10.2	10,434 ± 448^a^
	0.5	12.2 ± 0.3^b^	12.8 ± 0.32^c^	201.7 ± 2.1^b^	238.9 ± 2.4^c^	191.6 ± 2.6^c^	542.2 ± 10.2	10,903 ± 448^b^
	0.75	12.2 ± 0.3^b^	12.6 ± 0.32^b^	201.9 ± 2.1^b^	240.1 ± 2.4^d^	196.2 ± 2.6^d^	542.5 ± 10.2	11,083 ± 448^c^
	1	12.0 ± 0.3^a^	12.4 ± 0.32^a^	203.2 ± 2.1^c^	242 ± 2.4^e^	200.7 ± 2.6^e^	543.2 ± 10.2	11,392 ± 448^d^
	*p*-value	<0.01	<0.01	<0.01	<0.01	<0.01	0.96	<0.01
Additive	None	12.4 ± 0.3^c^	12.9 ± 0.3^c^	204.2 ± 2.4^c^	242.1 ± 2.4^c^	191.3 ± 2.8	541.1 ± 10.6	10,819 ± 657
	Monensin	12.2 ± 0.3^b^	12.7 ± 0.3^b^	200.8 ± 2.4^b^	238.7 ± 2.4^b^	191.4 ± 2.8	539.9 ± 10.6	10,852 ± 562
	Canola oil	12.1 ± 0.3^a^	12.6 ± 0.3^a^	198.1 ± 2.4^a^	236.1 ± 2.4^a^	190.6 ± 2.8	541.5 ± 10.6	10,815 ± 577
	*p*-value	<0.01	<0.01	<0.01	<0.01	0.12	0.54	0.43

*NCS, no carbon sequestration; WCS, with carbon sequestration; LW, live weight; DM, dry matter*.

1 *Baseline scenario*.

a, b, c, d, e *Within column, averages with different superscript differ significantly (p < 0.01)*.

Methane emissions represented between 83.7 and 83.3% of total GHG emissions, decreasing as supplementation increased (*p* < 0.01). The use of additives had an effect on the percentage of methane emissions (*p* < 0.01). The lowest percentage (83.3%) was obtained with canola oil. GHG emissions associated with pastures, supplementation, and direct N_2_O represented on average 6.5, 4.2, and 4.1% of total GHG emissions.

These results show that the effect of additives on methane emissions is greater than the increase in total emissions resulting from the inclusion of the supplement (with or without additives).

For the economic variables, supplementation had a significant effect on total costs, raising them by 8.2% at the highest level of supplementation (1 kg DM/calf/day) compared with the baseline scenario. However, no significant differences were observed between supplementation levels for operational and financial income (*p* < 0.01). The additive/calf supplementation interaction was not significant in any of the variables studied.

### Experimental Scenarios

**Figure 2:**
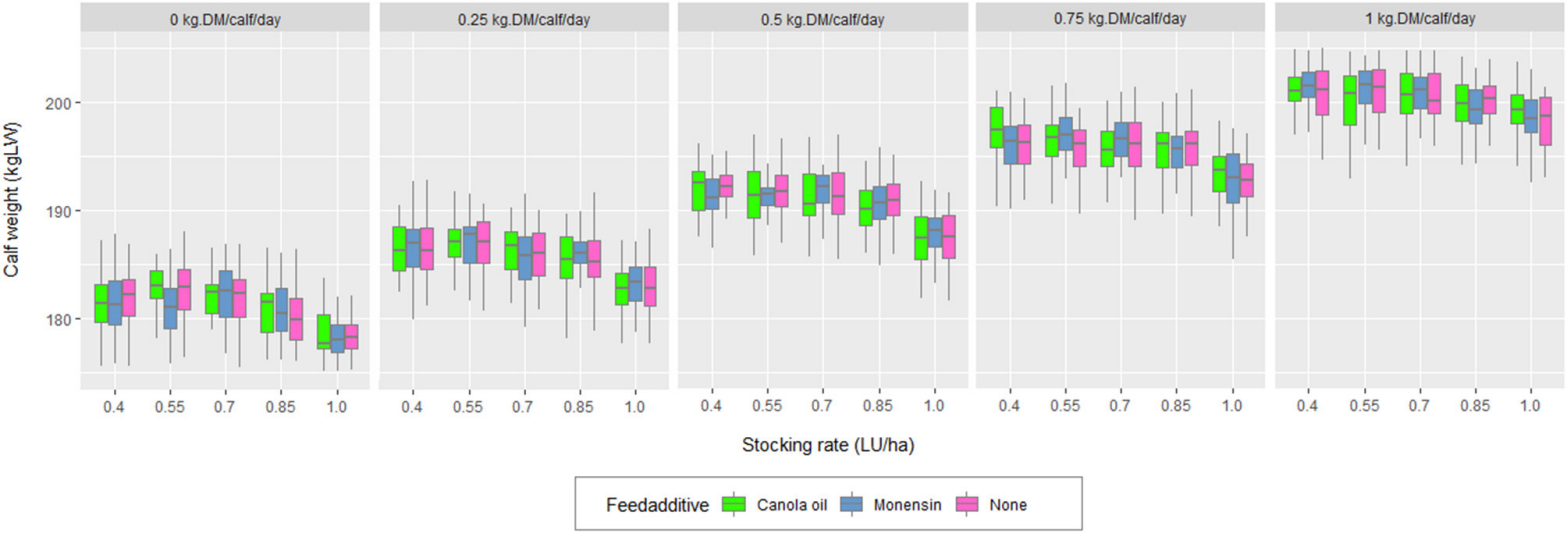
Calf weight according to stocking rate and level of calf supplementation.

A total of 65 scenarios were evaluated. Of these, 60 (five stocking rates, four levels of supplementation, and two additives + supplement without additive) included the supplementation of the calves. Table 3 displays the results for the three factors evaluated (stocking rate, calf supplementation, and additive). The variables stocking rate and calf supplementation had a significant effect on the weight of the calves and the total kg LW of calves weaned/sold (Figure 2). Regarding the economic variables, a significant effect of calf supplementation was observed on all the variables associated with costs and variable gross income (*p* < 0.01). The additive/calf supplementation interaction was not significant in any of the variables studied.

**Table 3:** Cost and income variables concerning calf supplementation (creep feeding) and additives (monensin and canola oil) in the baseline scenario (stocking rate of 0.7 LU/ha).

**Variables**	**Level**	**Total cost (US$)**	**Operational cost (US$)**	**Average total cost(US$/kg LW)**	**Average operational cost (US$/kg LW)**	**Total gross income(US$)**	**Financial income (US$)**	**Operational income (US$)**
Supplementation(kg DM/calf/day)	0	24,550 ± 164^a^	21,083 ± 157^a^	1.3 ± 0.03^a^	1.11 ± 0.03^a^	27,294 ± 657^a^	2,743 ± 638	6,210 ± 637
	0.25	25,083 ± 164^b^	21,615 ± 157^b^	1.3 ± 0.03^ab^	1.12 ± 0.03^ab^	27,705 ± 657^b^	2,622 ± 638	6,090 ± 637
	0.5	25,627 ± 164^c^	22,159 ± 157^c^	1.31 ± 0.03^bc^	1.13 ± 0.03^b^	28,241 ± 657^c^	2,614 ± 638	6,082 ± 637
	0.75	26,065 ± 164^d^	22,598 ± 157^d^	1.33 ± 0.03^d^	1.15 ± 0.03^c^	28,505 ± 657^d^	2,440 ± 638	5,907 ± 637
	1	26,574 ± 164^e^	23,107 ± 157^e^	1.32 ± 0.03^cd^	1.15 ± 0.03^c^	29,178 ± 657^e^	2,604 ± 638	6,071 ± 637
	*p*-value	<0.01	<0.01	<0.01	<0.01	<0.01	0.15	0.14
Additives	None	25,579 ± 164	22,112 ± 157	1.31 ± 0.03	1.14 ± 0.03	28,091 ± 657	2,512 ± 638	5,979 ± 637
	Monensin	25,572 ± 164	22,105 ± 157	1.31 ± 0.03	1.13 ± 0.03	28,202 ± 657	2,630 ± 638	6,097 ± 637
	Canola oil	25,588 ± 164	22,121 ± 157	1.31 ± 0.03	1.13 ± 0.03	28,261 ± 657	2,672 ± 638	6,140 ± 637
	*p*-value	0.77	0.76	0.51	0.51	0.18	0.19	0.18

*LW, live weight; DM, dry matter*.

a, b, c, d, e *Within column, averages with different superscript differ significantly (p < 0.01)*.

**Figure 3:**
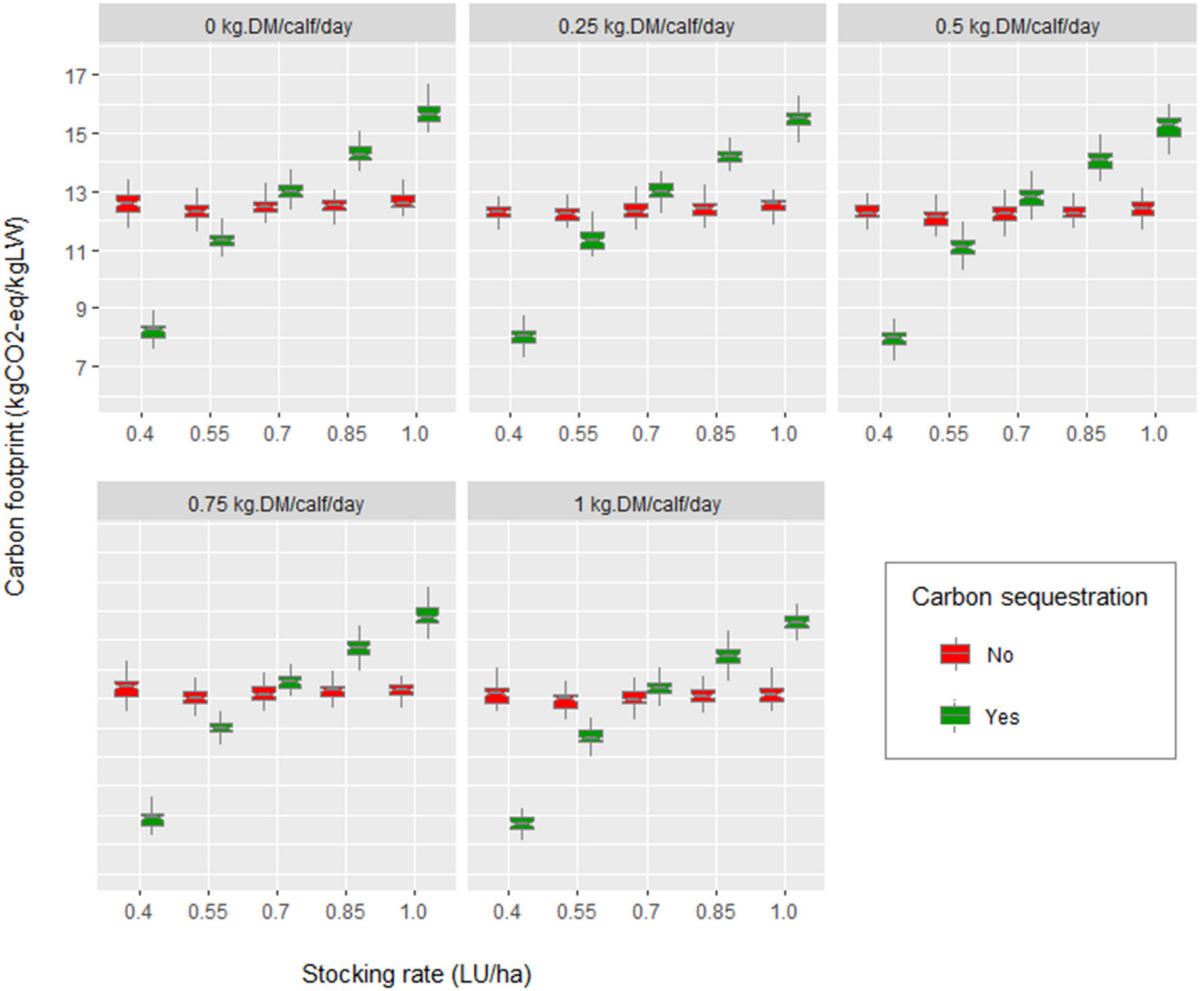
Effect of stocking rate (0.4, 0.55, 0.7, 0.85, and 1 LU/ha), calf supplementation (creep feeding), and carbon sequestration on carbon footprint (CF).

An interaction effect between calf supplementation and stocking rate was observed for CF, calf weight, and total production (*p* < 0.01) (Table 4). The quantification of carbon sequestration had a significant effect on the CF (*p* < 0.01) (Figure 3).

**Table 4:** Simulated CF, total emissions, and productive variables in relation to stocking rate (0.4, 0.55, 0.7, 0.85, and 1 LU/ha), calf supplementation (creep feeding), and additives (monensin and canola oil).

**Variable**	**Level**	**Carbon footprint** **(kg CO**_****2−eq****_**/kg LW)**		**Methaneemissions (tons of CO_**2−eq**_)**	**Totalemissions (tons of CO_**2−eq**_)**	**Calves weight (kg LW)**	**Cows weight (kg LW)**	**Total production (kg LW)**
		**NCS**	**WCS**					
Stocking rate (LU/ha)	0.4	12.4 ± 0.4^d^	8 ± 0.4^a^	114.3 ± 2.1^a^	135.9 ± 2.3^a^	191.5 ± 7.5^d^	547.0 ± 10.4^e^	6,085 ± 416^a^
	0.55	12.1 ± 0.4^a^	11.1 ± 0.4^b^	161.6 ± 2.8^b^	192.1 ± 3.1^b^	191.5 ± 7.0^cd^	544.6 ± 9.6^d^	8,743 ± 533^b^
	0.7	12.3 ± 0.4^b^	12.7 ± 0.4^c^	201.1 ± 3.7^c^	239.1 ± 3.9^c^	191.2 ± 7.5^c^	541.6 ± 10.5^c^	10,843 ± 607^c^
	0.85	12.3 ± 0.3^c^	14 ± 0.5^d^	242.4 ± 4.3^d^	288.4 ± 4.8^d^	190.3 ± 7.4^b^	535.7 ± 10.9^b^	13,236 ± 700^d^
	1	12.4 ± 0.4^d^	15.2 ± 0.6^e^	284.8 ± 5.4^e^	338.7 ± 5.9^e^	187.9 ± 7.8^a^	520.1 ± 10.2^a^	15,317 ± 818^e^
	*p*-value	<0.01	<0.01	<0.01	<0.01	<0.01	<0.01	<0.01
Supplementation (kg DM/calf/day)	0	12.5 ± 0.4^d^	12.5 ± 2.6^e^	198.7 ± 59.2^a^	235.7 ± 70.3^a^	180.6 ± 3.2^a^	537.6 ± 14.4	10,269 ± 3,131^a^
	0.25	12.4 ± 0.4^c^	12.4 ± 2.6^d^	199.8 ± 59.2^b^	237.2 ± 70.4^b^	185.6 ± 3^b^	537.4 ± 14.3	10,540 ± 3,150^b^
	0.5	12.3 ± 0.4^b^	12.2 ± 2.6^c^	200.8 ± 59.9^c^	238.9 ± 71.1^c^	190.6 ± 3^c^	537.4 ± 14.3	10,860 ± 3,251^c^
	0.75	12.2 ± 0.4^b^	12 ± 2.4^b^	201.9 ± 60.3^d^	240.3 ± 71.6^d^	195.4 ± 3^d^	537.4 ± 13.6	11,108 ± 3,396^d^
	1	12.1 ± 0.4^a^	11.8 ± 2.4^a^	202.9 ± 60.7^e^	242 ± 72.4^e^	200.2 ± 2.9^e^	539 ± 14.1	11,447 ± 3,490^e^
	*p*-value	<0.01	<0.01	<0.01	<0.01	<0.01	0.31	<0.01
Additives	None	12.5 ± 0.4^c^	12.3 ± 2.5^c^	204 ± 60.9^c^	242 ± 72.2^c^	190.4 ± 7.5	538.3 ± 13.7	10,848 ± 3,319
	Monensin	12.3 ± 0.4^b^	12.2 ± 2.6^b^	200.4 ± 59.6^b^	238.5 ± 71^b^	190.5 ± 7.6	537.5 ± 14.4	10,840 ± 3,300
	Canola oil	12.2 ± 0.4^a^	12 ± 2.6^a^	198.0 ± 59.0^a^	236 ± 70.3^a^	190.5 ± 7.6	537.6 ± 14.3	10,847 ± 3,315
	*p*-value	<0.01	<0.01	<0.01	<0.01	0.76	0.78	0.71
Total average		12.3 ± 0.4	12.2 ± 2.5	200.9 ± 59.8	238.9 ± 71.1	190.4 ± 7.6	537.9 ± 14.3	10,844 ± 3,305

*NCS, no carbon sequestration; WCS, with carbon sequestration; LW, live weight; DM, dry matter*.

a, b, c, d, e *Within column, averages with different superscript differ significantly (p < 0.01)*.

**Figure 4:**
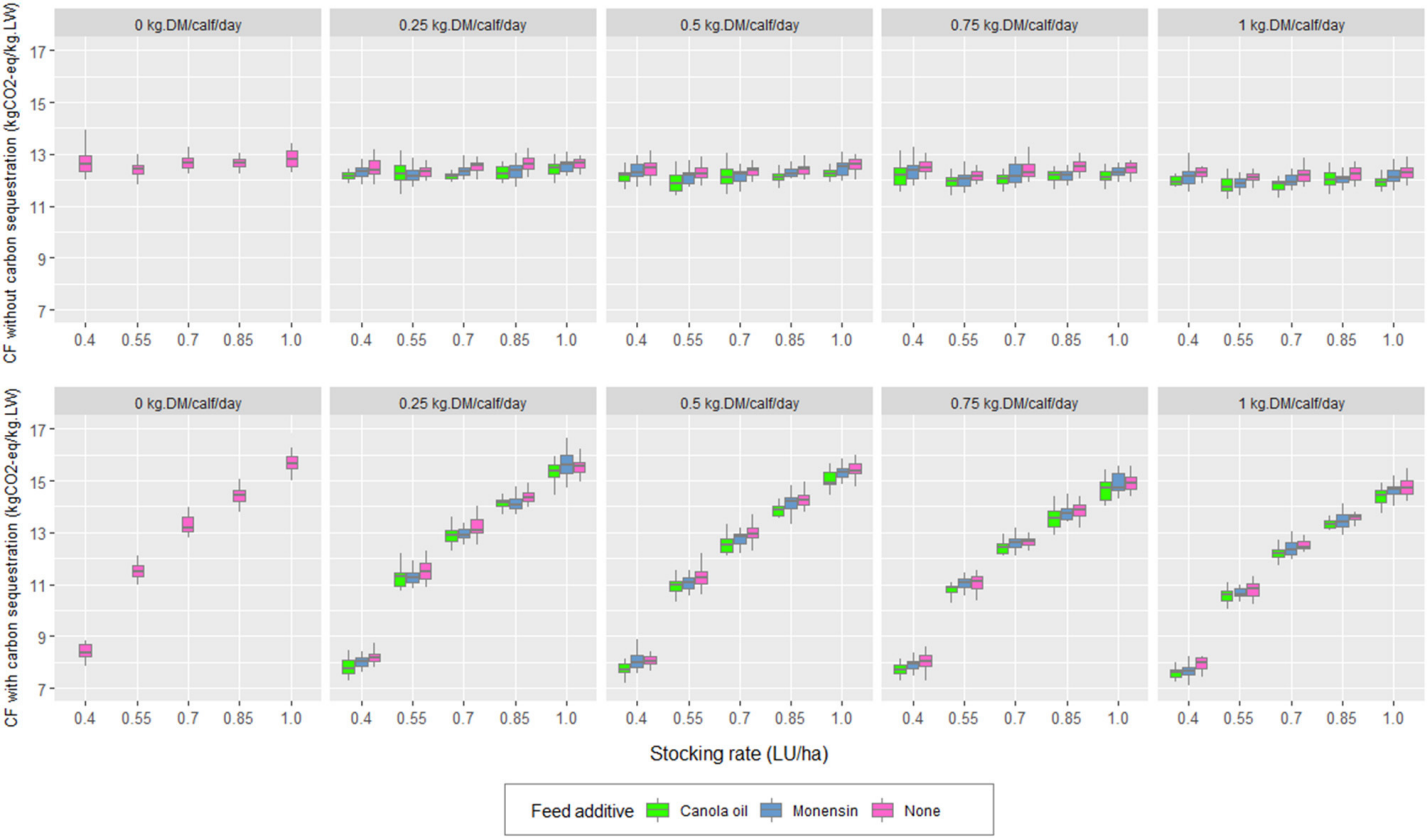
Effect of calf supplementation, additive stocking rate, and carbon sequestration on the carbon footprint (CF) of the simulated scenarios.

When carbon sequestration was not considered, CF averages decreased by 2.5 and 1.4% for canola oil and monensin, respectively. In the scenario with carbon sequestration, the average decreases in CF were 2.4% for canola oil and 1.3% for monensin (Table 3). Figure 4 shows the effect of the additives on the CF based on the calf supplementation and stocking rate in the estimates with and without consideration of carbon sequestration.

**Figure 5:**
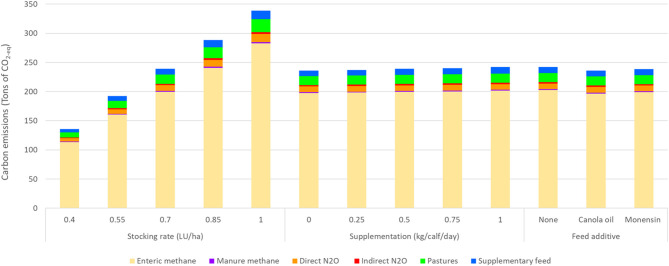
Total greenhouse gas (GHG) emissions according to stocking rate (0.4, 0.55, 0.7, 0.85, and 1 LU/ha), supplementation (creep feeding), and additive (monensin and canola oil).

Figure 5 illustrates the distribution of total GHG emissions concerning the evaluated variables in the simulated scenarios. In the systems evaluated, the items representing the highest percentages of GHG emissions corresponded to enteric methane emissions, pastures, supplementation, and direct N_2_O, with average percentages of 83.5, 6.6, 4.2, and 4.1%, respectively. Effects of calf supplementation, additive, and stocking rate were observed on the percentage distribution of total GHG emissions (*p* < 0.05).

For the economic variables (Table 5), results showed a significant effect for the supplementation, stocking rate, and the supplementation/stocking rate interaction on total cost, operational cost, total gross income, operational income, average total cost, average operational cost, and financial income (*p* < 0.01). The increase in stocking rate to 1 LU/ha increased total costs by 40.3% compared with the baseline scenario (0.7 LU/ha). However, due to the higher total production, increases of 39.6, 32.7, and 30.8% by total gross income, financial income, and operational income, respectively, were observed. The use of supplement generated an average increase of 1.9% in the total cost and 2.2% in the operational cost for each increase of 0.25 kg DM/calf/day. Given the increase in kilos sold due to supplementation, an increase occurred in the total income. Despite this, due to the increase in the average cost of production at higher supplementation levels, the operational income on average decreased by 0.3% product of supplementation. A significant effect of the stocking rate/additive interaction was also observed in the financial income and operational income (*p* < 0.01).

**Table 5:** Cost and income variables concerning supplementation and additives in the simulated scenarios.

**Variables**	**Level**	**Total cost (US$)**	**Operational cost (US$)**	**Average total cost (US$/kg LW)**	**Average operational cost (US$/kg LW)**	**Total gross income (US$)**	**Financial income (US$)**	**Operational income (US$)**
Stocking rate (LU/ha)	0.4	15,036 ± 404^a^	12,618 ± 404^a^	1.37 ± 0.05^c^	1.15 ± 0.04^d^	16,051 ± 616^a^	1,015 ± 493^a^	3,433 ± 492^a^
	0.55	20,767 ± 579^b^	17,777 ± 578^b^	1.31 ± 0.04^ab^	1.12 ± 0.03^a^	22,721 ± 817^b^	1,954 ± 587^b^	4,944 ± 585^b^
	0.7	25,581 ± 724^c^	22,113 ± 723^c^	1.31 ± 0.03^a^	1.13 ± 0.03^b^	28,150 ± 944^c^	2,569 ± 664^c^	6,037 ± 663^c^
	0.85	30,649 ± 871^d^	26,678 ± 870^d^	1.31 ± 0.03^a^	1.14 ± 0.03^c^	33,630 ± 1,134^d^	2,982 ± 755^d^	6,952 ± 753^d^
	1	35,908 ± 1,053^e^	31,418 ± 1,050^e^	1.32 ± 0.03^b^	1.15 ± 0.03^e^	39,316 ± 1,319^e^	3,409 ± 814^e^	7,899 ± 812^e^
	*p-*value	<0.01	<0.01	<0.01	<0.01	<0.01	<0.01	<0.01
Supplementation (kg/calf/day)	0	24,566 ± 7,002	21,098 ± 6,276	1.31 ± 0.04	1.12 ± 0.03	27,044 ± 7,905^a^	2,479 ± 1,096^c^	5,946 ± 1,732^c^
	0.25	25,099 ± 7,152	21,632 ± 6,427	1.32 ± 0.04	1.13 ± 0.03	27,479 ± 7,939^b^	2,381 ± 1,026^b^	5,847 ± 1,636^b^
	0.5	25,615 ± 7,314	22,148 ± 6,589	1.32 ± 0.04	1.14 ± 0.03	27,979 ± 8,103^c^	2,364 ± 1,028^ab^	5,831 ± 1,637^ab^
	0.75	26,088 ± 7,464	22,621 ± 6,739	1.33 ± 0.04	1.15 ± 0.03	28,386 ± 8,330^d^	2,298 ± 1,094^a^	5,765 ± 1,712^a^
	1	26,572 ± 7,624	23,104 ± 6,898	1.33 ± 0.04	1.15 ± 0.03	28,979 ± 8,548^e^	2,407 ± 1,116^bc^	5,875 ± 1,754^bc^
	*p*-value	<0.01	<0.01	<0.01	<0.01	<0.01	<0.01	<0.01
Additives	None	25,589 ± 7,344	22,122 ± 6,624	1.32 ± 0.04	1.14 ± 0.03	27,980 ± 8,213	2,391 ± 1,083	5,858 ± 1,710
	Monensin	25,587 ± 7,350	22,119 ± 6,627	1.32 ± 0.04	1.14 ± 0.03	27,955 ± 8,156	2,368 ± 1,056	5,835 ± 1,667
	Canola oil	25,588 ± 7,345	22,121 ± 6,624	1.32 ± 0.04	1.14 ± 0.03	27,987 ± 8,212	2,399 ± 1,082	5,866 ± 1,709
	*p*-value	0.95	0.92	0.79	0.81	0.54	0.55	0.56

a, b, c, d, e * Within column, averages with different superscript differ significantly (p < 0.01)*.

## Discussion

**Figure 6:**
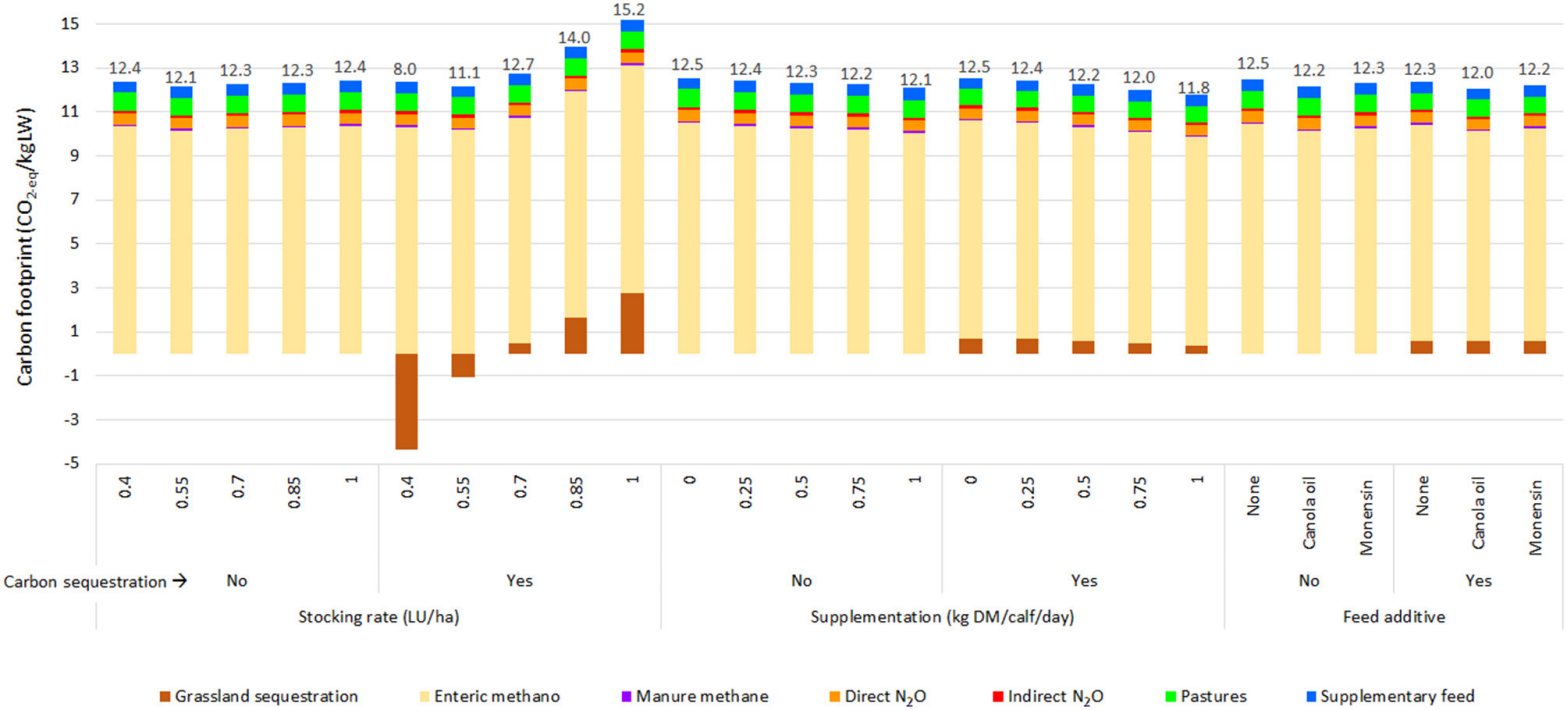
Carbon footprint (CF) and distribution of greenhouse gas (GHG) emissions in simulated scenarios.

The CF values obtained in this research were within the ranges described by Florindo et al. (52), Ogino et al. (57), and Crosson et al. (58), considering that the carcass yield of the common breeds in southern Chile is on average 56% (59). Similarly, the average weights gained by calves at 6 months in the scenarios without supplementation were similar to those reported by Catrileo et al. (9). As observed in Table 2, the CF tended to present higher values when the carbon sequestration was considered. This was confirmed in Figure 6, where part of the CF of the baseline scenario (stocking rate of 0.7 LU/ha) arose from the loss of carbon from the pasture and soil. Furthermore, the scenario with carbon sequestration showed that as the stocking rate increases, the CF increases. These results coincided with those reported by Toro-Mujica et al. (31) in extensive sheep production systems in rain-fed areas. As Sarkar et al. (60) point out, maintaining productivity continues to be a challenge for producers, implying maintaining a slightly higher than optimal system load. Thus, increasing the stocking rate without the corresponding increase in supplementation raises grazing pressure, decreasing the available photosynthetic material, the amount of litter with the potential to incorporate carbon into the soil, and the production of roots (61), resulting in less carbon sequestration to mitigate emissions. As Chen et al. (62) pointed out, improved grazing management regimes that allow the renewal of degraded grassland and promote carbon storage in the soil are required. On the other hand, no clear trend appeared to increase or decrease the CF, as the stocking rate increased in the scenarios without carbon sequestration. This meant that the stocking rate effect on the CF was dependent on the initial value of the stocking rate. However, the expected trend is to increase the CF when stocking rates exceed the level of equilibrium of the production system. This trend was shown slightly when the stocking rate was increased in the scenario without supplementation or with low supplementation levels (Figure 3). With regard to the distribution of total emissions, the percentage of enteric methane was higher than the 44% reported by Samsonstuen et al. (63), consistent with the lower use of inputs in the systems studied.

The significant effect of creep feeding on the weight of calves agreed with Carvalho et al. (64). The higher weight of calves with supplementation occurred because, in the supplementation months (summer/autumn), the pasture was in its reproductive stage, decreasing its nutritional value (65). Through a meta-analysis, Carvalho et al. (64) reported that the supplemental weight gain increases quadratically with supplementation (Figure 2). This trend was a consequence of the growth potential of the breed, of the potential feed intake, and the ability to substitute forage for a supplement without altering the functioning of the rumen. The greater gain in weaning weight resulted in reduced CF by kg of LW sold despite the increase in total emissions. In other words, the ratio between the extra production of emissions associated with supplementation and the extra kilos produced was less than the CF of the scenario without supplementation. Furthermore, an interaction between stocking rate and supplementation was observed. Thus, as the supplementation increased, the effect of the increase in the stocking rate on the weight of calves decreased (Figure 2). The interaction stocking rate/supplementation was also observed when the CF was analyzed considering the carbon sequestration. As the level of supplementation increased, the effect of the rise of the stocking rate on the CF decreased. Moreover, the weight of the cows was not affected by the level of supplementation of their calves, as reported by Carvalho et al. (64). This was because the calves decreased their pasture intake and not milk consumption as the level of supplementation rises (66). The pasture intake decreased with increasing supplementation levels (Figure 6). As the supplementation level increased, the proportion that carbon sequestration represented in the CF decreased, according to Crosson et al. (58). The additives incorporated into the supplementation, both monensin and canola oil, had slight but significant effects on decreasing the CF of the simulated systems (Table 3). Thus, the addition of canola oil into the supplement decreased the CF between 2 and 2.8% as the supplementation increased from 0.25 to 1 kg/calf/day. In the case of monensin, the decrease in the CF varied between 1.3 and 1.8%. In addition, the total enteric emissions showed decreases in percentages, ranging from 2.6 to 3.2% for canola oil and from 1.6 to 2.0% for monensin. The additives had a slight effect on the CF due to the fact that the supplement with the additive only represented between 6 and 21% of the total supplementation consumed and between 0.6 and 2.4% of the total feed consumed during the production cycle (for levels of 0.25 and 1 kg DM/calf/day, respectively). Then, a potential exists to reduce GHG emissions by incorporating these additives into the supplementation. For example, in the systems studied, this could be examined by increasing the period or level of supplementation in calves or by incorporating the additives into the supplementation of cows. This is an issue that needs to be analyzed in future research. In these studies, the secondary effects of increasing the level of supplementation need to be incorporated, since supplementation increases the mean retention time of feed in the rumen decreased, increased fractional passage rate, and decreased ruminal fermentation (67). This could alter the relative production of volatile fatty acids in the rumen and feed digestibility (68) and, thus, the efficiency of the feeding strategy. The incorporation of additives into the supplement had no effect on any of the economic variables examined. Three possible explanations emerged: a low level of additive per kg of feed, the periods and levels of supplementation evaluated, and the economic compensation of the cost of additive with the increase of the efficiency in the use of energy of the supplement. The increase in total costs due to supplementation did not give rise to lower financial or operational income. However, marginal profit decreased as the stocking rate increased. The above was consistent with the lower weight of cows and calves, and the subsequent lower milk production by decreasing DM availability in the pastures (69, 70).

## Conclusions

The model proposed explained the effect of feed and management strategies on GHG emissions and production costs. The incorporation of additives into the supplementation of calves decreased the CF in the analyzed production system without increasing production costs. The most significant effects on CF were obtained with the highest levels of supplementation. Although the magnitude of the additives' effects was low, it can be increased as the proportion of the feed with additives is increased. In the first instance, including an additive to the cows' supplement emerges as an alternative. The stocking rate used in the baseline scenario was very close to the optimal level of the production system. This shows how extensive systems are managed to reach a point of equilibrium. In this way, the use of new production strategies in the cow–calf production systems requires an adaptation period and a comprehensive adjustment in the factors of production. The adaptation period, as well as the time required to reach an optimal CF, can be reduced using simulation models. For the simulated WCS baseline scenario (0.7 LU/ha), the CF (−5.1%) can be decreased and the financial income (5.8%) increased by supplementing calves with 0.75 kg DM/day without additives. Furthermore, by supplementing calves with 0.25 kg DM/day of monensin, a decrease in CF (−2.9%) and an increase in financial income (8.4%) were obtained. When an increase in stocking rate (0.85 LU/ha) was simulated, a slight increase in the CF (1.9%) allowed a significant increase (15.3%) in the financial income of the farm through the supplementation of calves with 0.75 kg DM/day and canola oil as additives.

Considering the international agreements to reduce GHG emissions, using a simulation model to evaluate feeding or management strategies in cow–calf production systems before its implementation on the farm appears to be an alternative that deserves further exploration. The secondary supplementation effects on rumen function and milk quality need to be examined in future simulation models.

## Data Availability Statement

The raw data supporting this study are available on request to the corresponding author.

## Author Contributions

PT-M: model design, experimentation, statistical analysis, data interpretation, and wrote the manuscript.

## Conflict of Interest

The author declares that the research was conducted in the absence of any commercial or financial relationships that could be construed as a potential conflict of interest.
